# Supplementary material for: Modeling and Analysis of Morphology of Injection Molding Polypropylene Parts Induced by In-Mold Annealing
Source: Polymers (Basel). 2022 Dec 1;14(23):5245. doi: 10.3390/polym14235245 (PMC9740916; doi:10.3390/polym14235245)
Supplement: Supplementary file 1 [file polymers-14-05245-s001.zip › polymers-2040165-supplementary.pdf]

Supplementary File S1: Model for the crystallinity evolution.

A complete model to describe the crystallinity evolution of the α-phase in the considered iPP was recently proposed [S1]. In the model the α-phase is composed of two types of crystals: spherulites and shish-kebabs. The effect of flow on both crystals' evolution and the dependence of material parameters upon crystallinity are accounted for in the proposed model.

The evolution into isotropic structures was described adopting the Kolmogoroff's equation

$$\varphi_0 = \frac{4}{3}\pi \int_{-\infty}^{t} \dot{N}_s(s) \left[\int_s^t G_s(u)du\right]^3 ds \qquad \text{(S1)}$$

where $\dot{N}_s$ is the nucleation rate of the spherulites, G_s is the growth rate of the spherulites.

The fibrils were assumed having a cylindrical shape whose growth was described also by a Kolmogoroff's equation accounting for the geometrical anisotropy:

$$\psi_0 = 2\pi \int_{-\infty}^{t} \dot{N}_f(s) \int_s^t G_f(u) \left[\int_u^t G_{lat}(p)dp\right]^2 du\, ds \qquad \text{(S2)}$$

$\dot{N}_f$ represents the nucleation rate of the fibrils, G_f is the fibrils axial growth rate, in other words the growth rate along the flow direction, and G_{lat} represents the growth rate in the direction transverse to the flow, which is assumed to be equal to the spherulites growth rate, G_s, under the same flow condition.

The effect of flow on the crystallinity evolution was accounted for both fibrils and spherulites evolution. These effects are considered through the molecular orientation assuming that the polymer behavior is similar to the behavior of two beads connected by a non-bendable spring. The Maxwell's model accounts for such a behavior through the molecular conformation tensor $\boldsymbol{A}$.

$$\frac{DA}{Dt} - (\boldsymbol{\nabla u})^T \cdot \boldsymbol{A} - \boldsymbol{A} \cdot (\boldsymbol{\nabla u}) = -\frac{\mathrm{A}}{\lambda(A)} + (\boldsymbol{\nabla u})^T + (\boldsymbol{\nabla u}) \qquad \text{(S3)}$$

where $\boldsymbol{\nabla u}$ is the velocity gradient and λ is the relaxation time assumed to be dependent upon the molecular orientation. A scalar measure of the stretch that the molecules experience by the effect of flow during the process, Δ, is given by the largest difference between the eigenvalues of the molecular conformation tensor $\boldsymbol{A}$. In simple shear Δ can be expressed as

$$\Delta = \sqrt{{A_{xx}}^2 + 4A_{xy}^2} \qquad \text{(S4)}$$

where x is the flow direction, y is the direction transversal to the flow. Equation 5 describes the dependence of the relaxation time upon the molecular stretch parameter, Δ:

$$\lambda(T,P,\chi,\Delta) = \frac{\lambda_0 \cdot \alpha(T,P,\chi_c)}{1+(a\Delta)^b} \qquad \text{(S5)}$$

where a and b are material parameters, λ_0 is the relaxation time at rest. The dependence of λ upon temperature, pressure and total crystallinity, X_c, and all the material parameters are reported elsewhere [S1]. Particularly, the shift factor α, the same used for the viscosity, describes the dependence of the relaxation time on pressure, temperature and crystallinity.

$$\alpha(T,P,\chi_c) = 10^{\frac{-A1(T-A3-D3P)}{A2+T-A3}} \exp(180\,\chi_c^2) \qquad \text{(S6)}$$

where the material parameters are: λ_0= 14 s, *D3*= 0.18 K/MPa, *A1*=2.5, *A2*=301.4 K, *A3*=503 K. The relaxation time decreases when pressure decreases; thus, a faster relaxation process is expected in the presence of low pressures.

For spherulites evolution, the growth rate is expressed by the Hoffman-Lauritzen equation.

$$G_s(T(t), P, \Delta) = G_0 exp\left(-\frac{U}{R(T-T_\infty(P))}\right) exp\left(-\frac{K_g(T+T_m(P,\Delta))}{2T^2(T_m(P,\Delta)-T)}\right) \quad \text{(S7)}$$

where G_0, K_g, T_∞, and U/R are material constants (G_0 = 1380 cm/s, K_g = 371381 K^2, T_∞ = 198.46 K, and $\frac{U}{R}$ = 751.6 K). The melting temperature in quiescent conditions and atmospheric pressure, $T_{m,0}$, is 467.54 K. Two shift factors were used for melting and glass temperatures to account for the effect of pressure on the crystallization kinetics. Particularly, melting and glass transition temperatures increase of 1.6 and 3.1 K, respectively, for an increase of pressure of 10 MPa. Moreover, experiments [S2-S4] suggested that the effect of flow on spherulitic kinetics is twofold. Firstly, the melting temperature T_m increases with the molecular stretch with respect to its quiescent value [S2]. Secondly, an additional nucleation rate with respect to the quiescent heterogenous nucleation was considered. Such an additional nucleation rate was correlated to the excess of radial growth rate (the difference between the spherulites growth rate in flow condition and the growth rate in quiescent condition) by effect of flow with respect to the quiescent value.

The effect of flow on fibrils crystalline functions was identified in [S1]. Growth rates of fibrils in equation 2 were considered dependent upon the molecular stretch. In particular, the lateral growth rate, the growth in the direction transversal to the flow, is considered equal to the spherulites growth rate i.e.

$$G_{lat}(T(t), \Delta) = G_s(T(t), \Delta) \quad \text{(S8)}$$

The fibrils axial growth occurs if the molecular stretch assumes values higher than a threshold, above which the axial growth rate follows a power-law. The axial growth rate, G_f, is given in equation 9:

$$G_f = A_0 \Delta^b \quad \text{(S9)}$$

where $A_0 = 4\ 10^{-8}$ cm/s, and $b = 5$.

Also, for the description of the fibrils evolutions a dependency of nucleation rate upon the flow is considered. In particular, the correlation between fibrils nucleation rate and excess of axial growth rate is the same adopted for the spherulites kinetics

The overall relative crystallinity degree ξ of the alpha crystalline phase, which is the predominant phase in the injection molded parts made of the iPP grade adopted in this work, is given in equation 10

$$-\ln(1-\xi) = \psi_0 + \varphi_0 \quad \text{(S10)}$$

where φ_0 and ψ_0 are described by equations 1 and 2, respectively.

References

1. Speranza, V.; Liparoti, S.; Volpe, V.; Titomanlio, G.; Pantani, R. Modelling of morphology development towards spherulites and shish–kebabs: Application to isothermal flow-induced crystallization experiments on isotactic polypropylene. *Polymer (Guildf).* **2020**, *196*, 122459, DOI: 10.1016/j.polymer.2020.122459.

2. Pantani, R.; Speranza, V.; Titomanlio, G. Effect of flow-induced crystallization on the distribution of spherulite dimensions along cross section of injection molded parts. *Eur. Polym. J.* **2017**, *97*, 220–229, DOI: 10.1016/j.eurpolymj.2017.10.012.
3. De Santis, F.; Pantani, R.; Titomanlio, G. Effect of shear flow on spherulitic growth and nucleation rates of polypropylene. *Polymer (Guildf).* **2016**, *90*, 102–110, DOI: 10.1016/j.polymer.2016.02.059.
4. Pantani, R.; Coccorullo, I.; Volpe, V.; Titomanlio, G. Shear-Induced Nucleation and Growth in Isotactic Polypropylene. *Macromolecules* **2010**, *43*, 9030–9038, DOI: 10.1021/ma101775h.
